# A defined subunit vaccine that protects against vector-borne visceral leishmaniasis

**DOI:** 10.1038/s41541-017-0025-5

**Published:** 2017-08-21

**Authors:** Malcolm S. Duthie, Lais Pereira, Michelle Favila, Kimberly A. Hofmeyer, S. Jim Reed, Sonia Metangmo, Shannon Townsend, John D. Laurance, Alessandro Picone, Ayesha Misquith, Faria Hossain, Prakash Ghosh, Md Anik Ashfaq Khan, Jeffery Guderian, H. Remy Bailor, Hong Liang, Julie Vergara, Fabiano Oliveira, Randall F. Howard, Shaden Kamhawi, Dinesh Mondal, Rhea N. Coler, Jesus G. Valenzuela, Steven G. Reed

**Affiliations:** 1 Infectious Disease Research Institute, 1616 Eastlake Avenue East, Suite 400, Seattle, WA 98102 USA; 2 Vector Molecular Biology Section, Laboratory of Malaria and Vector Research, National Institute of Allergy and Infectious Diseases, National Institutes of Health, Rockville, MD 20852 USA; 3 International Center for Diarrhoeal Diseases Research, Laboratory Sciences Division, Dhaka, Bangladesh

## Abstract

Vaccine development for vector-borne pathogens may be accelerated through the use of relevant challenge models, as has been the case for malaria. Because of the demonstrated biological importance of vector-derived molecules in establishing natural infections, incorporating natural challenge models into vaccine development strategies may increase the accuracy of predicting efficacy under field conditions. Until recently, however, there was no natural challenge model available for the evaluation of vaccine candidates against visceral leishmaniasis. We previously demonstrated that a candidate vaccine against visceral leishmaniasis containing the antigen LEISH-F3 could provide protection in preclinical models and induce potent T-cell responses in human volunteers. In the present study, we describe a next generation candidate, LEISH-F3+, generated by adding a third antigen to the LEISH-F3 di-fusion protein. The rationale for adding a third component, derived from cysteine protease (CPB), was based on previously demonstrated protection achieved with this antigen, as well as on recognition by human T cells from individuals with latent infection. Prophylactic immunization with LEISH-F3+formulated with glucopyranosyl lipid A adjuvant in stable emulsion significantly reduced both *Leishmania infantum* and *L. donovani* burdens in needle challenge mouse models of infection. Importantly, the data obtained in these infection models were validated by the ability of LEISH-F3+/glucopyranosyl lipid A adjuvant in stable emulsion to induce significant protection in hamsters, a model of both infection and disease, following challenge by *L. donovani*–infected *Lutzomyia longipalpis* sand flies, a natural vector. This is an important demonstration of vaccine protection against visceral leishmaniasis using a natural challenge model.

## Leishmaniasis: Improved vaccine candidate demonstrates protection

A research collaboration between the United States and Bangladesh has improved its own vaccine candidate and demonstrated it to be effective. The vaccine’s target, visceral leishmaniasis, is a deadly parasitic disease transmitted via the bites of sandflies. The collaboration, led by Steven Reed of the United States’ Infectious Disease Research Institute, previously developed a vaccine that presents two parasite-derived proteins (antigens) to a host’s immune system, allowing it to build a defense. The new vaccine candidate contains three antigens and has shown to confer disease resistance to hamsters who were subsequently exposed to infected sandflies. This is the first time a vaccine for visceral leishmaniasis has stood up in tests where the host has been infected through natural methods, which inspires confidence in the vaccine’s capacity to work in the wild.

## Introduction

The leishmaniases are a group of vector-borne diseases transmitted by phlebotomine sand flies that regurgitate then inoculate *Leishmania* parasites during blood meals. Perhaps 350 million people in endemic areas are at risk of infection and subsequent development of leishmanaisis in one form or another.^1, 2^ Visceral leishmaniasis (VL) results from infection with either *L. infantum* or *L. donovani* and can be fatal in a substantial subset of patients infected by *L. donovani* if left untreated. Even when treated, a substantial subset of patients (5–50% depending upon region) will develop the sequelae known as post kala-azar dermal leishmaniasis (PKDL), in which parasites in the skin, often forming visible lesions, are available for uptake and transmission by sand flies. Humans are the dominant reservoir for VL caused by *L. donovani*, and an effective vaccine could interrupt transmission in a sustainable VL elimination strategy. Attempts to control *L. donovani* infections in the Indian subcontinent have mainly centered on improved chemotherapy and reducing insect vector populations to decrease transmission, and both approaches have contributed to advancing VL elimination in the region. Despite very significant progress in each of these areas, it is generally agreed that a safe and effective vaccine will be needed to achieve broad elimination goals across Asia, Africa, and Latin America. This is also true for *L. infantum* infection, for which dogs and other mammals can serve as reservoirs. An effective vaccine could be used not only to prevent progression of *Leishmania* infection to disease but also to prevent or reduce infection and thereby reduce transmission.

While many defined or semi-defined parasite antigens have been evaluated as vaccine components in pre-clinical models of leishmaniasis, the important impact of vector-derived proteins (e.g., those derived from salivary glands during parasite inoculation) on infection and immunity is also now evident.^3, 4^ Sand flies rely on the salivary anti-hemostatic components to acquire a blood meal, but a number of immuno-modulatory activities have also been reported.^4^ Such anti-inflammatory activities may facilitate parasite transmission and establishment of infection. This provides a complication to overcome in vaccine development and, to date, it has been unclear whether needle challenge can provide adequate predictive information regarding vaccine efficacy. Hamsters inoculated with *Leishmania* during bites from infected sand flies have recently emerged as a more physiologically relevant and stringent system for the evaluation of anti-*Leishmania* vaccines.^5, 6^

We recently advanced a single chimeric di-fusion protein (LEISH-F3; produced by contiguous expression of the *nh* and *smt* genes) through a phase 1 clinical study (ref. 7 and unpublished data). Our vaccine development scheme consists of combining select antigens with adjuvant formulations appropriate for use in humans. In this report, we modified the LEISH-F3 construct by adding a truncated cysteine protease B (CPB; C) to render a tri-fusion protein, designated as LEISH-F3+, with potential for broader antigen recognition in *Leishmania*-affected regions. The truncation involved the targeted removal of amino-acid sequences from CPB that were homologous to human sequences; removing these sequences did not alter the protective capacity of the CPB protein. With appropriate adjuvant formulation in GLA-SE, the LEISH-F3+ protein provided robust immune responses similar to those obtained with LEISH-F3. In this study, we evaluated vaccine-induced protection in experimental systems initially using needle challenge with cultured *Leishmania* parasites, followed by evaluation in a newly developed natural challenge model. Altogether, our data support clinical development of LEISH-F3+ for field evaluation as a VL vaccine.

## Results

### Immune recognition of a truncated CPB

**Fig. 1:**
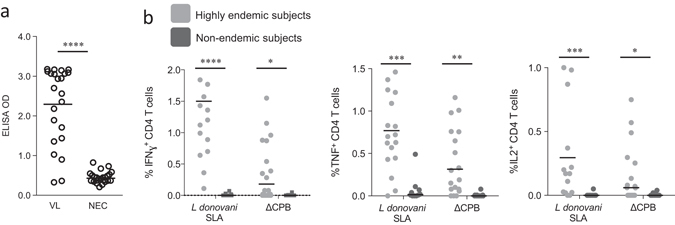
Recognition of truncated cysteine protease B protein (ΔCPB) by *L. donovani-*affected individuals. In **a**, serum antibodies against ΔCPB were measured by ELISA in samples from VL patients (*VL*; *n* = 23) and non-endemic control individuals (*NEC*; *n* = 24). Each point represents the response of each individual sample with *black bars* indicating the mean OD for each group. In **b**, peripheral blood mononuclear cells from subjects living in either a highly endemic (*n* = 20) or non-endemic (*n* = 10) region were incubated with *L. donovani* SLA or ΔCPB prior to analyses by flow cytometry to identify CD4 T cells producing IFN-γ, TNF or IL-2, respectively. Each point represents the response of each individual sample with *black bars* indicating the mean for each group. Positive responses were determined as having optical densities (*OD*) or cell populations greater than the mean plus 3 s.e.m of that observed with NEC. Statistical significance was calculated by Kolmogorov–Smirnov test, *p < 0.05, ***p < 0.001, and ****p < 0.0001

*Leishmania* cysteine protease B (CPB) has shown promise as a VL vaccine candidate antigen in mouse and dog infection models.^8, 9^ Genome analyses indicated segments with significant homology of *cpb* with human genes, however, so to minimize any likelihood of generating anti-host responses, we deleted amino acids 1–153 of the *cpb* gene, generating a truncated recombinant variant of CPB (ΔCPB) devoid of potential self-reactive epitopes (Table 1). The retention of antigenicity was demonstrated by showing that VL patients, unlike non-endemic controls, had serum antibodies against ΔCPB protein (91.3%; Fig. 1a). Furthermore, many individuals living in a VL endemic region and suggested to have prior parasite exposure indicated by T-cell responses to soluble *L. donovani* antigen (SLA), had CD4 T cells that produced interferon gamma (IFN-γ), interleukin (IL)-2, and/or tumor necrosis factor (TNF) following incubation with ΔCPB (12 of 20; 11 of 20; and 17 of 20; respectively; Fig. 1b), further demonstrating T-cell antigenicity of the truncated protein and supporting its potential use in a vaccine.

**Table 1:** Attributes of the components within the LEISH-F3 or LEISH-F3+ fusion proteins

Name	Source	Species conservation	Size (kD)	Potential HLA-binding motifs	Description	Notes and references
Nucleoside hydrolase	*L. donovani*	100% *L. infantum*	36	None	Main immunogenic component of the fucose-mannose ligand (*FML*) complex of *L. donovani*	NH, formulated with saponin, protects mice against VL^19^
	*—*	96% *L. major*	*—*	*—*	*—*	FML, formulated with QuilA saponin, protects dogs against VL^20^
	*—*	93% *L. mexicana*	*—*	*—*	*—*	*—*
	*—*	92% *L. tropica*	*—*	*—*	*—*	*—*
	*—*	84% *L. braziliensis*	*—*	*—*	*—*	*—*
Sterol-24-c-methyl-transferase	*L. infantum*	99% *L. donovani*	40	None	Involved in the biosynthesis of ergosterol (a membrane sterol in yeast and *Leishmania* spp.)	Protects mice against VL and CL when vaccinated subcutaneously with MPL-SE^21, 22^
	*—*	97% *L. major*	*—*	*—*	*—*	*—*
	*—*	94% *L. mexicana*	*—*	*—*	*—*	*—*
	*—*	86% *L. braziliensis*	*—*	*—*	*—*	*—*
Delta cysteine protease “B”	*L. infantum*	99% *L. donovani*	34	None	Stage-regulated cathepsin-L-like cysteine protease (divergent carboxy-terminus aa#154-443)	Full length gene protects mice and dogs against VL when vaccinated using DNA prime and protein boost with CpG ^9^
	*—*	68% *L. major*	*—*	aa1–aa153: sequence removed for vaccine subunit	*—*	*—*
	*—*	73% *L. mexicana*	*—*	*—*	*—*	*—*
	*—*	83% *L. tropica*	*—*	*—*	*—*	*—*
	*—*	57% *L. braziliensis*	*—*	*—*	*—*	*—*

### Construct and characterization of LEISH-F3+

**Fig. 2:**
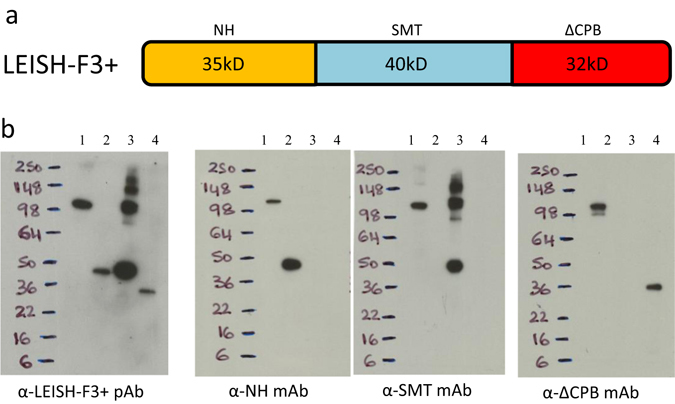
Construction and characterization of the LEISH-F3+ fusion protein. In **a**, a cartoon depiction of the LEISH-F3+ fusion protein is shown. In **b**, LEISH-F3+ fusion protein was characterized by immunoblot. Recombinant LEISH-F3+ (*lane 1*), Nucleoside hydrolase (*NH*; *lane 2*), Sterol-24-c-methyl-transferase (*SMT*; *lane 3*) or truncated cysteine protease B protein (Δ*CPB*; lane 4); were loaded at 100 ng each lane into gels. Blots were developed with mouse polyclonal or monoclonal antibodies as indicated, derived from the same original gel. The cartoon depiction of the fusion protein is the authors own rendering

We recently demonstrated that LEISH-F3, a vaccine antigen candidate incorporating the nucleoside hydrolase (NH) and sterol-24-c-methyltransferase (SMT) antigens, afforded protection against experimental *Leishmania* infection and safely generated antigen-specific responses in phase I clinical trials (ref. 7 and unpublished data). The present report extends these findings to the next generation candidate, LEISH-F3+, generated by adding ΔCPB to LEISH-F3 to create LEISH-F3+ (Fig. 2a). The immunogenicity and antigenicity of LEISH-F3+ was confirmed by immunoblotting with an affinity purified polyclonal serum against LEISH-F3+ and recognition of a band at the expected molecular weight (Fig. 2b). The anti-LEISH-F3+ sera also recognized the individual NH, SMT, and ΔCPB proteins at the appropriate sizes and, in return, LEISH-F3+ was recognized by monoclonal antibodies against each component antigen (Fig. 2b).

**Fig. 3:**
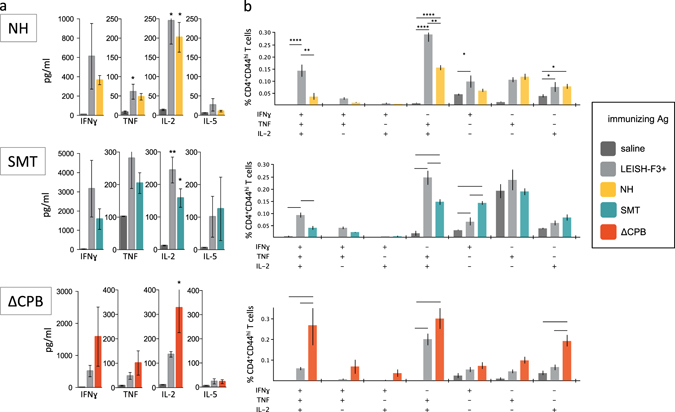
Immune recognition of each component within LEISH-F3+. C57BL/6 mice were injected a total of three times with 1 μg LEISH-F3+ protein, or a molar equivalence of the individual NH, SMT and ΔCPB proteins alone, formulated with GLA-SE. One month after the final immunization spleens were removed to prepare single cell suspensions (*n* = 3). In **a**, cells were incubated with antigen indicated in the box for 4 days then cytokine content in the culture supernatant determined by ELISA. In **b**, cells were subjected to flow cytometry to identify antigen-experienced CD4 T cells and the cytokine protection profile (various combinations of IFN-γ, IL-2, or TNF). Data are shown as mean and s.e.m, three mice per group. Data are representative of results obtained in two similar experiments

To further assess immunogenicity of the LEISH-F3+ antigen, we evaluated the immune responses induced by the fusion protein relative to those induced by immunization with the single components. As expected, when formulated in GLA-SE, all of the antigens generated antigen-specific Th1-skewed immune responses, characterized by production of IFN-γ, TNF, and IL-2, but lower levels of IL-5 (Fig. 3a). Pluripotent antigen-specific CD4 T cells producing either all three of the Th1 cytokines examined, or combinations thereof, were readily detected (Fig. 3b). Similar results were obtained when cells from mice immunized with LEISH-F3+ were compared with cells from mice immunized with an equimolar mixture of the three component antigens approximating the fusion protein (Supplementary Fig. 1). Altogether, these data indicate that the immune reactivity of each component is retained following immunization with the LEISH-F3+ construct.

### LEISH-F3+/GLA-SE protects mice against VL-causing *Leishmania* species

**Fig. 4:**
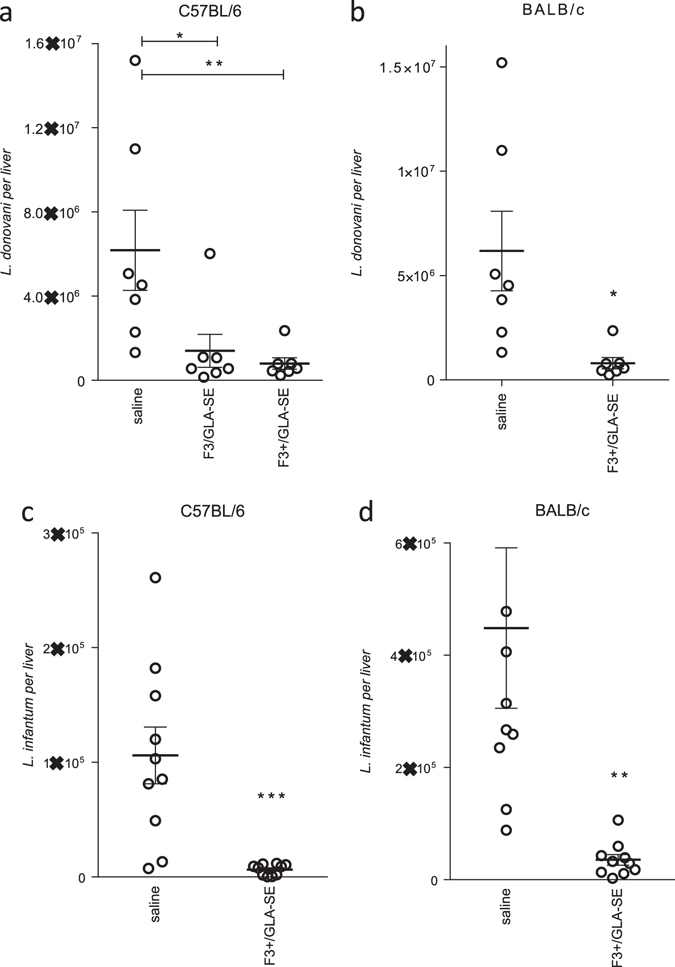
Immunization with LEISH-F3+ reduces *Leishmania* infection. Mice were subcutaneously injected a total of three times with 5 μg protein formulated with GLA-SE, then 1 month after the final immunization were infected by intravenous injection of *Leishmania* spp. promastigotes. Livers were removed 1 month after inoculation and parasite burdens were determined by qPCR. In **a** and **b**, C57BL/6 and BALB/c mice, respectively, were infected with *L. donovani*. In **c** and **d**, C57BL/6 and BALB/c mice, respectively, were infected with *L. infantum*. Each point represents the burden of each individual mouse, with the *bars* indicating the mean and s.e.m for each group with 7–10 mice per group. Data are representative of results obtained with each protein in 2–3 independent experiments

To assess the protective capacity of the LEISH-F3+ vaccine candidate, and of the composite components, *L. donovani* challenge studies were performed in mice. Parasite burdens following challenge of mice immunized with a formulated tri-fusion containing NH, SMT, and the full length CPB component (NSC) were significantly reduced relative to unimmunized mice (Supplememtary Fig. 1; 94.9%). Immunization with LEISH-F3+ construct, containing the truncated CPB (ΔCPB) also resulted in significant reduction in *L. donovani* burdens in the liver (Supplementary Fig. 1; 95.2%), indicating that the deleted portion of CPB was not required for protection. When C57BL/6 mice were immunized with either the previously described NS (LEISH-F3) or LEISH-F3+ formulated in GLA-SE, *L. donovani* burdens in the liver were reduced to a similar extent (Fig. 4a; 77.3% and 87.1% reduction relative to unimmunized animals, respectively). BALB/c mice were similarly protected against *L. donovani* infection by immunization with LEISH-F3+/GLA-SE (Fig. 4b). Thus, in this inbred mouse model, LEISH-F3+ antigen elicits protective responses at least equivalent to an antigen (LEISH-F3) that has advanced to a clinical study.

While infection with *L. donovani* causes VL cases in the Indian subcontinent and East Africa, *L. infantum* is the causative agent of VL in the Mediterranean basin, central Asia, and the Americas. To evaluate if LEISH-F3+/GLA-SE immunization could also protect against experimental *L. infantum* infection, C57BL/6 mice and BALB/c mice were immunized and infected with *L. infantum*. When liver burdens were assessed 4 weeks later, we determined that LEISH-F3+/GLA-SE immunization significantly reduced parasite numbers compared to those measured in saline-treated mice (Fig. 4c, d). Taken together, these data demonstrate that LEISH-F3+/GLA-SE elicits protection against experimental VL in genetically distinct mouse strains and *Leishmania* species.

### Protection against *L. longipalpis* sand fly-transmitted *L. donovani* infection

**Fig. 5:**
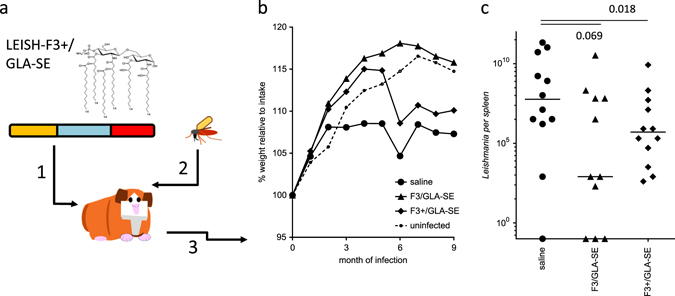
Immunization with LEISH-F3+/GLA-SE reduces parasite burden in hamsters infected with *L. donovani* during sand fly blood meals. In **a**, hamsters were injected a total of three times with 5 ug protein formulated with GLA-SE (step 1), then 1 month after the final immunization were infected by bites of *L. donovani*-infected sand flies (step 2), then monitored (step 3). In **b**, hamster weights were checked just prior to exposure to sand flies then monitored throughout the infection phase of the experiment. Each point depicts the group mean at the given time. In **c**, spleens were removed 9 months after parasite inoculation and burdens were determined by qPCR. Each point represents the burden of each individual animal, with the *bars* indicating the median of 11–12 hamsters per group. Statistical significance was calculated by Wilcoxon-signed rank sum analysis. Data are representative of results obtained in two similar experiments. The images are the authors own renderings

Natural, vector-borne infection of hamsters represents a stringent model for the evaluation of vaccines against *L. donovani*. Unlike needle challenge with purified parasites, the sand fly vector strongly influences infection by introducing immune modulatory proteins in the saliva at the same time as the parasite.^4^ We, therefore, immunized hamsters with either LEISH-F3 or LEISH-F3+ (each formulated with GLA-SE) prior to inoculating them with physiologically relevant quantities of *L. donovani* via bites from infected *L. longipalpis* sand flies (Fig. 5a). Immunization was confirmed by the presence of circulating antigen-specific antibodies prior to infection (data not shown). Weight gain was observed in all hamsters within the first 2 months after challenge after which the weight of the unimmunized, but infected hamsters plateaued (Fig. 5b and Supplementary Fig. 3). This contrasted with the weight of the hamsters in the immunization groups which, over the first 4–5 months after infection, demonstrated weight gains comparable to those observed in control uninfected hamsters (Fig. 5b). After allowing the infection to propagate for 9 months, spleens were harvested and parasite burdens determined. While hamsters immunized with either LEISH-F3/GLA-SE or LEISH-F3+/GLA-SE had markedly reduced *L. donovani* burdens when compared to untreated infected hamsters (90.2% and 97.7%, respectively), a statistically significant reduction was observed only for LEISH-F3+/GLA-SE immunized animals (Fig. 5c). Burdens in 55% of the LEISH-F3/GLA-SE and 67% of the LEISH-F3+/GLA-SE immunized hamsters were lower than those observed in all but 2 of the 12 unvaccinated hamsters. Thus, LEISH-F3+/GLA-SE immunization protected against *Leishmania* infection in a model of natural infection. In total, the combined results from both of the vaccine candidates support the overall antigen/adjuvant selection as an approach for vaccine implementation in an overall VL elimination strategy.

## Discussion

Vaccination has the potential to contribute to an overall VL elimination strategy by not only protecting against infection/ disease, but also by impacting infectious reservoirs to reduce *Leishmania* transmission. An effective vaccine will be particularly important for control of VL in Africa where the distribution of the vectors over vast areas reduces the feasibility of vector control. There are at least two ways in which a vaccine could be applied in an elimination strategy, both to prevent disease and transmission. The first is to immunize uninfected or recently infected individuals to prevent infection and/or disease progression (post exposure prophylaxis). A second approach is to apply a vaccine after chemotherapy of VL to prevent the development of PKDL, as PKDL patients are undoubtedly a source of parasites to sand flies. The leishmaniases are neglected diseases with those affected generally living in poverty and for these reasons investments in vaccine development have been scarce. It is, therefore, critical that efforts to demonstrate preclinical efficacy are maximized in the most relevant models possible. Data support the approach of using natural infection of susceptible animals as a more rigorous way to evaluate vaccine candidates.^5, 6^ However, until now, no natural challenge model has been available to evaluate VL vaccine candidates.

Because of the ability of defined adjuvants to direct as well as enhance immune responses, we have focused on a protein/adjuvant approach for developing vaccine candidates that could be applied either prior to or after infection. We developed a tri-fusion vaccine antigen candidate, LEISH-F3+, comprised of three parasite antigens each with demonstrated protective capacity. Expression of these as a recombinant polyprotein has led to a vaccine component that can be produced inexpensively in countries where leishmaniasis is prevalent, including India and Brazil. Of the available adjuvants shown to enhance the T-cell responses that are considered critical for protection against leishmaniasis, TLR4 agonists are by far the most advanced and, indeed, are the only TLR agonists in approved vaccines.

Data obtained in mouse models using needle challenge indicated that immunization with LEISH-F3+/GLA-SE protected against both experimental *L. donovani* and *L. infantum* infection, the causative agents of VL. It has become apparent that sand fly components may impact subsequent *Leishmania* infection, however, and in some cases, protection induced by vaccination in needle challenge models was not reproduced when sand fly challenge was used.^4, 10^ It is evident that achieving efficacy in a natural challenge model of *Leishmania* represents the highest bar available for the validation of vaccine candidates. To extend these results, we developed a natural challenge model of VL whereby parasite challenge was achieved via *L. longipalpis* sand fly bites. To achieve this, we exposed hamsters to infected sand flies over several days, mimicking the situation that likely occurs in VL-endemic regions and providing a highly stringent model with which to evaluate any preventative measures. While high levels of parasites were detected in the spleens of unimmunized hamsters, significantly lower burdens were recovered from the spleens of hamsters immunized with LEISH-F3+/GLA-SE.

We recently demonstrated that the LEISH-F3 vaccine candidate, containing two protein antigens, not only protected mice against needle challenge but induced potent immune responses in human volunteers.^7^ To increase epitope diversity, without introducing sequences with human homology in the vaccine, we created a truncated variant of the *cpb* gene in which amino acids 1–153 were removed to render a product devoid of any predicted self-reactive epitopes. The resulting polyprotein, LEISH-F3+, is thus devoid of human homology. Interestingly, immunization with LEISH-F3+/GLA-SE generated higher percentages of NH- and SMT-specific multifunctional memory CD4 T cells capable of producing combinations of IFN-γ, TNF, and IL-2 than immunization with an equivalent amount of the single antigens (NH or SMT alone)+ GLA-SE. Vaccine-generated multifunctional CD4 T cells have been correlated with protection against various intracellular pathogens, including *Leishmania*.^11, 12^ Furthermore, recognition of each subunit protein after immunization with the fusion indicates a lack of immune dominance between the antigenic components, and the immunological profiles observed in mice correlated with protective responses in both mice and hamsters.

We have previously demonstrated that specific sand fly salivary proteins can confer protection against leishmaniasis, including both CL and VL.^13, 14^ This led us to propose that a sand fly salivary protein should be a component of a vaccine against leishmaniasis with the hypothesis that a combination of *Leishmania* antigens with a sand fly salivary antigen will probably produce a broader, stronger response against the powerful initial challenge of a *Leishmania*-infected sand fly. In this work, we show that the *Leishmania*-derived LEISH-F3+ fusion protein can protect against VL initiated by a sand fly challenge. A combination of LEISH-F3+ with a salivary protein, possibly LJM19,^15^ may produce an even more robust vaccine for use in *Leishmania*-endemic regions.

In this study, the relevance of the LEISH-F3+/GLA-SE vaccine candidate was validated in a natural challenge model. Although both antigens, LEISH-F3 and LEISH-F3+, hold promise as vaccine components, the F3+ antigen, based on human immune responses and antigen diversity, as well as significant protection in the natural challenge model described herein, will be prioritized for further clinical development. The adjuvant selection also proved to be critical to achieve protection in both needle and natural challenge models. In this regard, formulation of either antigen with another advanced adjuvant containing the TLR4L monophosphoryl lipid A (MPL) in a liposomal/QS21 formulation afforded no protection with either antigen (unpublished data).

In conclusion, our data identify a chimeric fusion protein incorporating the N, S and ΔCPB (a truncated version of the CPB antigen) that, when formulated with GLA-SE, induced protective immunity against *Leishmania* infection in a natural challenge model, as well as in syringe challenge in mice. Thus, we have confirmed findings from a previous study on experimental CL,^16^ that challenge models involving needle infection with cultured promastigotes can be predictive of efficacy in a natural challenge model. While of direct application to the control of VL, our study may also prove relevant for the evaluation of vaccines against other vector-borne diseases.

## Materials and methods

### Ethics statement

Individuals were enrolled within the Mymensingh and Rajshahi districts, Bangladesh, with each participant, or parent or guardian, providing written informed consent for the collection of samples and subsequent analysis. The investigation was approved by the icddr,b and Rajshahi Medical College ethical review committee. Mouse experiments were conducted in accordance with procedures approved by the IDRI Animal Care and Use Committee. Hamster experiments were conducted in accordance with procedures approved by the NIAID Animal Care and Use Committee. Animals were distributed randomly in different cages with group sizes calculated to allow minimal animal usage required to provide proper statistical analysis based on estimates of anticipated variation of rodent models of *Leishmania* infection and on prior experience with the experimental system. The investigators were not blinded to analysis.

### Recombinant proteins

Recombinant proteins were cloned and expressed in *E. coli* as previously described.^17, 18^ The fusion protein was constructed by aligning the individual gene sequences as a single product. Affinity-purified protein fractions were analyzed by sodium dodecyl sulfate-polyacrylamide gel electrophoresis (SDS-PAGE) and quantified using the BCA protein assay (Pierce, Rockford, IL). Endotoxin levels were measured by Limulus Amebocyte Lysate QCL-1000 assay (Lonza Inc., Basel, Switzerland) and were all <100 EU/mg protein. Bioinformatic analyses to determine homology of the proteins between *Leishmania* species were conducted using Basic Local Alignment Search Tool (BLAST; https://blast.ncbi.nlm.nih.gov/Blast.cgi). Analyses to identify stretches greater than seven amino acids in length that were identical to human proteins were conducted to reveal potential self-epitopes.

### Antigen-specific antibody responses of VL patients

High-binding 384-well ELISA plates (Corning, MA) were coated overnight at 4°C with 50 µl/well of recombinant protein diluted in carbonate buffer. The next day, plates were washed with 0.1% Tween-20 in PBS and 200 µl of blocking buffer (PBS+ 1% BSA) added to each well for 2 h at room temperature. After blocking, plates are washed five times before serum samples diluted in 0.1% Tween-20+ 0.1% BSA in PBS was added at 50 µl/well and incubated for 30 min at room temperature. After incubation, plates were washed and 50 µl/well of peroxidase labeled-anti-human IgG (Life Technologies CA) in serum diluent was added. Plates were incubated for 30 min at room temperature, and then washed as previously described. To reveal reactions 100 µl of TMB SureBlue Peroxidase Substrate (Kirkegaard and Perry Laboratories, Gaithersburg, MD) was added to each well for 15 min at room temperature, after which the reaction was stopped by adding 1 N H_2_SO_4_. Plates were read within 10 min of reaction stoppage at optical density (OD) 450 nm, using 570 nm as the reference wavelength on a SpectraMax plate reader (Molecular Devices, CA).

### Western blotting

Samples for immunoblotting were prepared by suspending parasites in SDS sample buffer followed by boiling 5 min. Samples containing 5 × 10^5^ parasites were separated by SDS-PAGE and blotted on a polyvinylidene difluoride membrane (Invitrogen, Carlsbad, CA). Sera from rabbits immunized six times with 250 μg of either recombinant protein formulated in Freund’s incomplete adjuvant were used as the primary antibody. The membrane was then probed with HRP-conjugated F(ab)2-fragment donkey anti-rabbit IgG (Jackson ImmunoResearch Laboratories, Inc., West Grove, PA) and developed using a Chemiluminescent Super Sensitive HRP Substrate Kit (BioFX Laboratories, Owings Mills, MD).

### Mice and immunizations

Female C57BL/6 mice (purchased from Charles River Laboratories, Wilmington, MA) were maintained in specific pathogen-free conditions and in accordance with animal procedures approved by the IDRI institutional animal care and use committee. Mice entered experiments at 6–8 weeks of age and were immunized by subcutaneous injection of recombinant protein formulated with adjuvant at the base of the tail. Vaccines were prepared to provide a total of 5 μg/dose protein and 5 μg/dose GLA-SE in a total volume of 0.1 ml. When multiple proteins were used simultaneously, they were mixed prior to use to provide molar equivalence of each within a total dose of 5 μg. Mice were injected a total of three times at 3-week intervals. Experiments were conducted in a single blind manner with investigators unaware of group status.

### Analyses of mouse antibodies

Blood was collected, serum prepared and antigen-specific antibody responses were analyzed by ELISA for total IgG, as well as IgG2 and IgG1 isotypes. Briefly, ELISA plates (Nunc, Rochester, NY) were coated with 1 μg/ml antigen in 0.1 M bicarbonate buffer and blocked with 0.1% BSA-PBS. Then, in consecutive order and following washes in PBS/Tween, serially diluted serum samples, anti-mouse IgG, IgG1 or IgG2c-HRP (all Southern Biotech, Birmingham, AL) and ABTS-H_2_O_2_ (Kirkegaard and Perry Laboratories, Gaithersburg, MD) were added to the plates. Plates were analyzed at 405 nm (EL_X_808, Bio-Tek Instruments Inc, Winooski, VT). Endpoint titer was determined as the last OD value greater than a threshold determined by sera from unimmunized mice.

### Determining antigen-specific cell responses

One month after the final immunization, spleens were removed and single cell suspensions prepared. Mononuclear cells were enumerated using a ViaCount assay with a PCA system (Guava Technologies, Hayward, CA). Cells were cultured at 2 × 10^5^ cells per well in duplicate in a 96-well plate (Corning Incorporated, Corning, NY) in RPMI-1640 supplemented with 5% heat-inactivated FCS and 50,000 Units penicillin/streptomycin (Invitrogen), in the presence of 10 μg/ml protein. Culture supernatants were harvested after 4 days and cytokine content determined by ELISA, according to the manufacturer’s instructions (eBioscience, San Diego, CA).

### Infection of mice by needle inoculation

Mice were infected by injection of 1 × 10^6^ *L. donovani* (MHOM/SD/00/1S-2D) or *L. infantum* (MHOM/BR/82/BA-2) into the retro-orbital sinus. Parasites had been routinely passed through Syrian golden hamsters to generate virulent amastigote and promastigote stocks in M199 medium. Livers were harvested, homogenized and parasite burden calculated by limiting dilution assay or real-time PCR. DNA was extracted from homogenate using QIAmp DNA mini kits (Qiagen) and quantified using Nanodrop ultraviolet–visible spectrophotometer (ND-1000). *L. donovani* DNA was detected using primers for L42486 (forward, 5′- GCGACGTCCGTGGAAAGAA-3′; and reverse, 5′- GGCGGGTACACATTAGCAGAA-3′) with FAM reporter sequence (5′- CAACGCGTATTCCC-3′) that detects a 203-bp genomic repeat region specific to *Leishmania* species (NCBI Blastn). Mouse Gapdh FAM (Life Technologies) was used as an internal reference control. The number of parasite per µl of DNA was determined by extrapolating the crossing points (Cps) of each sample against a standard curve generated with known quantities of parasites, then burdens expressed as parasites per organ.

### Infection of hamsters during sand fly blood meals

Experimental procedures were reviewed, approved and conducted in accordance with the NIAID Animal Care and Use Committee. Male 4-week old Golden Syrian hamsters (Hsd Han TM-AURA strains) were purchased from Harlan Laboratories (Indianapolis, IN) and housed under pathogen-free conditions at the National Institute of Allergy and Infectious Diseases (NIAID). *Lutzomyia longipalpis* (*L. longipalpis*) sand flies, Jacobina strain, were reared at the Laboratory of Malaria and Vector Research, NIAID. Female 2- to 4-day-old *L. longipalpis* females were infected by artificial feeding on defibrinated rabbit blood (Spring Valley Laboratories, Sykesville, MD) containing 5 × 10^5^/ml *L. donovani* supplemented with 30 μl penicillin/streptomycin (10,000 units penicillin/10 mg streptomycin) was added per ml of blood for 3 h in the dark. Fully blood-fed flies were separated and maintained at 26 °C with 75% humidity and were provided 30% sucrose. Sand flies were used on day 10 after infection for transmission to hamsters. To assess the *L. donovani* infection status of the sand flies, 10–16 flies were washed and each midgut was separately macerated with a pestle in an eppendorf tube containing 50 μl of PBS. The parasite content within 10 μl of the solution was calculated by counting in a hemocytometer using a microscope, and percentage of metacyclics per midgut were determined. Flies (40–50) with mature infections were applied to both ears of each hamster through a meshed surface of vials held in place by custom-made clamps. All hamsters were anesthetized by intraperitoneal injection of ketamine (100 mg/kg) and xylazine (10 mg/kg) during exposure to the sand flies, which were allowed to feed for 2 h, in the dark for each session. Each hamster was exposed to bites from infected *L. longipalpis* sand flies for a total of three sessions conducted over 3 consecutive days. As a qualitative measure of transmission, the feeding status of all sand flies allowed to bite on the hamsters was evaluated. Each hamster received an average of 10 ± 2.8 infected bites per transmission.

### Parasite load in hamsters

Hamster spleens were aseptically removed and weighed, then a piece of each spleen was cut, weighed and macerated before being subjected to DNA extraction according to the manufacturer’s protocol (DNeasy Blood Tissue kit; Qiagen) to generate material to calculate parasite burden by real-time PCR. Primers called *Leishmania* kinetoplast minicircles (Primer-1 (JW11), 5′-CCTATTTTACACCAACCCCCAGT-3′; Primer-2 (JW12), 5′-GGGTAGGGGCGTTCTGCGAAA-3′), targets that are present in region conserved of different species of *Leishmania*, were used. Fluorescent reactions were performed using SYBR Green I (Molecular Probes, Eugene, OR) and the number of parasite per µl of DNA was determined by extrapolating the Cps of each sample against a standard curve generated with known quantities of parasites. Burdens were then expressed as parasites per organ.

### Statistical analyses

For data generated with human samples, statistical significance was calculated by Kolmogorov–Smirnov test, using GraphPad Prism 7.02 (GraphPad Prism Inc., La Jolla, CA). For data generated using mouse samples, *p*-values were determined using Student’s *t*-test also conducted in GraphPad Prism. For data generated with hamster samples, Wilcoxon signed rank sum analysis was conducted with MS Excel (Microsoft, Redmond, WA). Statistical significance was considered when the *p*-values were <0.05.

### Data availability

The underlying data reported in this paper are available from the corresponding author upon reasonable request.

## Electronic supplementary material

Supplementary Figure 1

Supplementary Figure 2

Supplementary Figure 3

Supplementary Figure 4

## References

[1] Alvar J (2012). Leishmaniasis worldwide and global estimates of its incidence. PLoS One.

[2] Bern C, Maguire JH, Alvar J (2008). Complexities of assessing the disease burden attributable to leishmaniasis. PLoS Negl. Trop. Dis..

[3] Andrade BB, de Oliveira CI, Brodskyn CI, Barral A, Barral-Netto M (2007). Role of sand fly saliva in human and experimental leishmaniasis: current insights. Scand. J. Immunol..

[4] Abdeladhim M, Kamhawi S, Valenzuela JG (2014). What’s behind a sand fly bite? The profound effect of sand fly saliva on host hemostasis, inflammation and immunity. Infect Genet. Evol..

[5] Aslan H (2013). A new model of progressive visceral leishmaniasis in hamsters by natural transmission via bites of vector sand flies. J. Infect. Dis..

[6] Fiuza JA (2016). Intradermal immunization of Leishmania donovani centrin knock-out parasites in combination with salivary protein LJM19 from sand fly vector induces a durable protective immune response in hamsters. PLoS Negl. Trop. Dis..

[7] Coler RN (2015). From mouse to man: safety, immunogenicity and efficacy of a candidate leishmaniasis vaccine LEISH-F3+GLA-SE. Clin. Transl. Immunol..

[8] Rafati S, Zahedifard F, Nazgouee F (2006). Prime-boost vaccination using cysteine proteinases type I and II of Leishmania infantum confers protective immunity in murine visceral leishmaniasis. Vaccine.

[9] Rafati S (2005). Protective vaccination against experimental canine visceral leishmaniasis using a combination of DNA and protein immunization with cysteine proteinases type I and II of L. infantum. Vaccine.

[10] Peters, N. C. *et al*. Evaluation of Recombinant Leishmania Polyprotein Plus Glucopyranosyl Lipid A Stable Emulsion Vaccines against Sand Fly-Transmitted Leishmania major in C57BL/6 Mice. *J. Immunol*. **189**, 4832–4841 (2012). 10.4049/jimmunol.1201676 PMC3596879 23045616

[11] Darrah PA (2007). Multifunctional TH1 cells define a correlate of vaccine-mediated protection against Leishmania major. Nat. Med..

[12] Seder RA, Darrah PA, Roederer M (2008). T-cell quality in memory and protection: implications for vaccine design. Nat. Rev. Immunol..

[13] Oliveira F, Lawyer PG, Kamhawi S, Valenzuela JG (2008). Immunity to distinct sand fly salivary proteins primes the anti-Leishmania immune response towards protection or exacerbation of disease. PLoS Negl. Trop. Dis..

[14] Gomes R (2008). Immunity to a salivary protein of a sand fly vector protects against the fatal outcome of visceral leishmaniasis in a hamster model. Proc. Natl Acad. Sci. USA.

[15] Asojo OA (2017). Structure of SALO, a leishmaniasis vaccine candidate from the sand fly Lutzomyia longipalpis. PLoS Negl. Trop. Dis..

[16] Oliveira F (2015). A sand fly salivary protein vaccine shows efficacy against vector-transmitted cutaneous leishmaniasis in nonhuman primates. Sci. Transl. Med..

[17] Duthie MS (2007). Use of protein antigens for early serological diagnosis of leprosy. Clin. Vaccine Immunol..

[18] Duthie MS (2008). Antigen-specific T-cell responses of leprosy patients. Clin. Vaccine Immunol..

[19] Aguilar-Be I (2005). Cross-protective efficacy of a prophylactic Leishmania donovani DNA vaccine against visceral and cutaneous murine leishmaniasis. Infect. Immun..

[20] Borja-Cabrera GP (2002). Long lasting protection against canine kala-azar using the FML-QuilA saponin vaccine in an endemic area of Brazil (Sao Goncalo do Amarante, RN). Vaccine.

[21] Goto Y, Bogatzki LY, Bertholet S, Coler RN, Reed SG (2007). Protective immunization against visceral leishmaniasis using Leishmania sterol 24-c-methyltransferase formulated in adjuvant. Vaccine.

[22] Goto Y (2009). Leishmania infantum sterol 24-c-methyltransferase formulated with MPL-SE induces cross-protection against L. major infection. Vaccine.

