# Supplementary material for: A defined subunit vaccine that protects against vector-borne visceral leishmaniasis
Source: NPJ Vaccines. 2017 Aug 21;2:23. doi: 10.1038/s41541-017-0025-5 (PMC5627294; doi:10.1038/s41541-017-0025-5)
Supplement: Supplementary file 1 — Supplementary Figure 1 [file 41541_2017_25_MOESM1_ESM.pdf]

Duthie et al., Suppl Figure 1

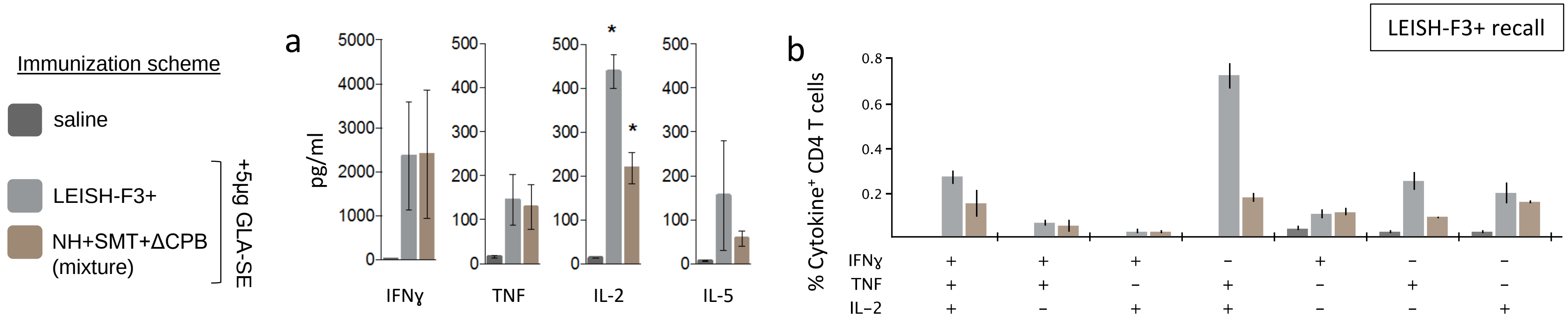

Supplementary Figure 1. Immune recognition of LEISH-F3+. C57BL/6 mice were injected a total of 3 times with either LEISH-F3+ or an equimolar mixture of the component antigens, each formulated with GLA-SE, then one month after the final immunization spleens were removed to prepare single cells suspensions (n = 3). In (a), cells were incubated with LEISH-F3+ for 4 days then cytokine content in the culture supernatant determined by cytokine bead array. In (b), cells were subjected to flow cytometry to identify antigen-experienced CD4 T cells and the cytokine protection profile (various combinations of IFNγ, IL-2 or TNF). Data are shown as mean and s.e.m, 3 mice per group. Data are representative of results obtained in 2 similar experiments.
