# Supplementary material for: A defined subunit vaccine that protects against vector-borne visceral leishmaniasis
Source: NPJ Vaccines. 2017 Aug 21;2:23. doi: 10.1038/s41541-017-0025-5 (PMC5627294; doi:10.1038/s41541-017-0025-5)
Supplement: Supplementary file 2 — Supplementary Figure 2 [file 41541_2017_25_MOESM2_ESM.pdf]

Duthie et al., Suppl Figure 2

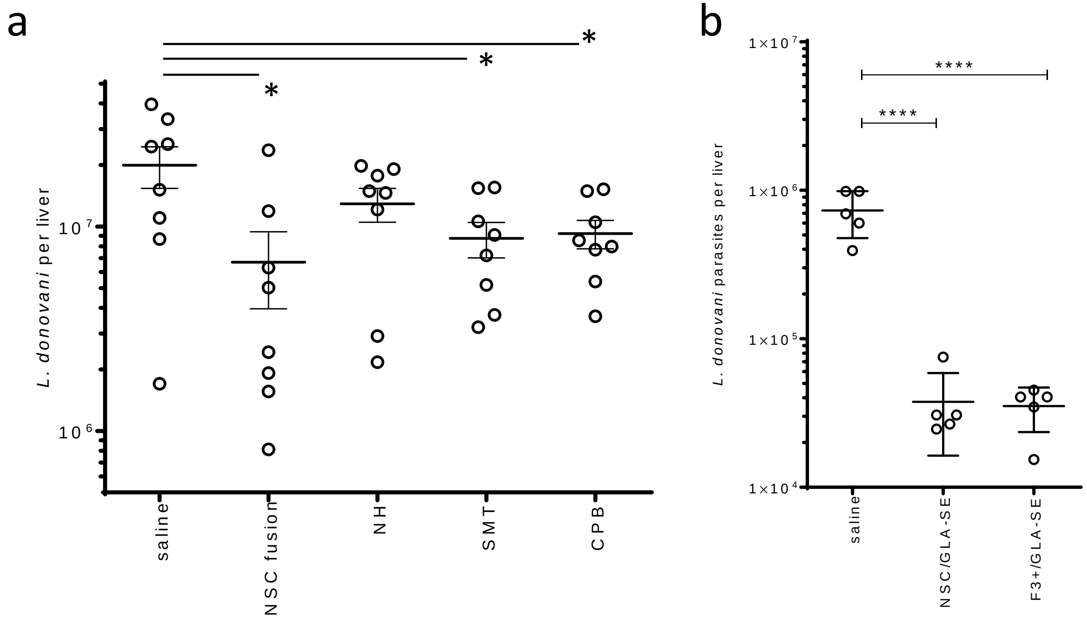

Supplementary Figure 2. Immunization with LEISH-F3+ reduces *Leishmania* infection. C57BL/6 mice were subcutaneously injected a total of 3 times with 5µg protein formulated with GLA-SE, then one month after the final immunization were infected by intravenous injection of *L. donovani* promastigotes. Livers were removed one month after inoculation and parasite burdens were determined by qPCR. In (a) mice were immunized with either a fusion incorporating full length CPB, (NSC) or the NH, SMT and CPB components. In (b), mice were immunized with either the NSC or LEISH-F3+ fusion protein. Each point represents the burden of each individual mouse, with the bars indicating the mean and s.e.m for each group with 7 mice per group. Data are representative of results obtained in 2 similar experiments. * = p-value < 0.05, ****= p-value < 0.0001.
