# Supplementary material for: A defined subunit vaccine that protects against vector-borne visceral leishmaniasis
Source: NPJ Vaccines. 2017 Aug 21;2:23. doi: 10.1038/s41541-017-0025-5 (PMC5627294; doi:10.1038/s41541-017-0025-5)
Supplement: Supplementary file 3 — Supplementary Figure 3 [file 41541_2017_25_MOESM3_ESM.pdf]

Duthie et al., Supplementary Figure 3

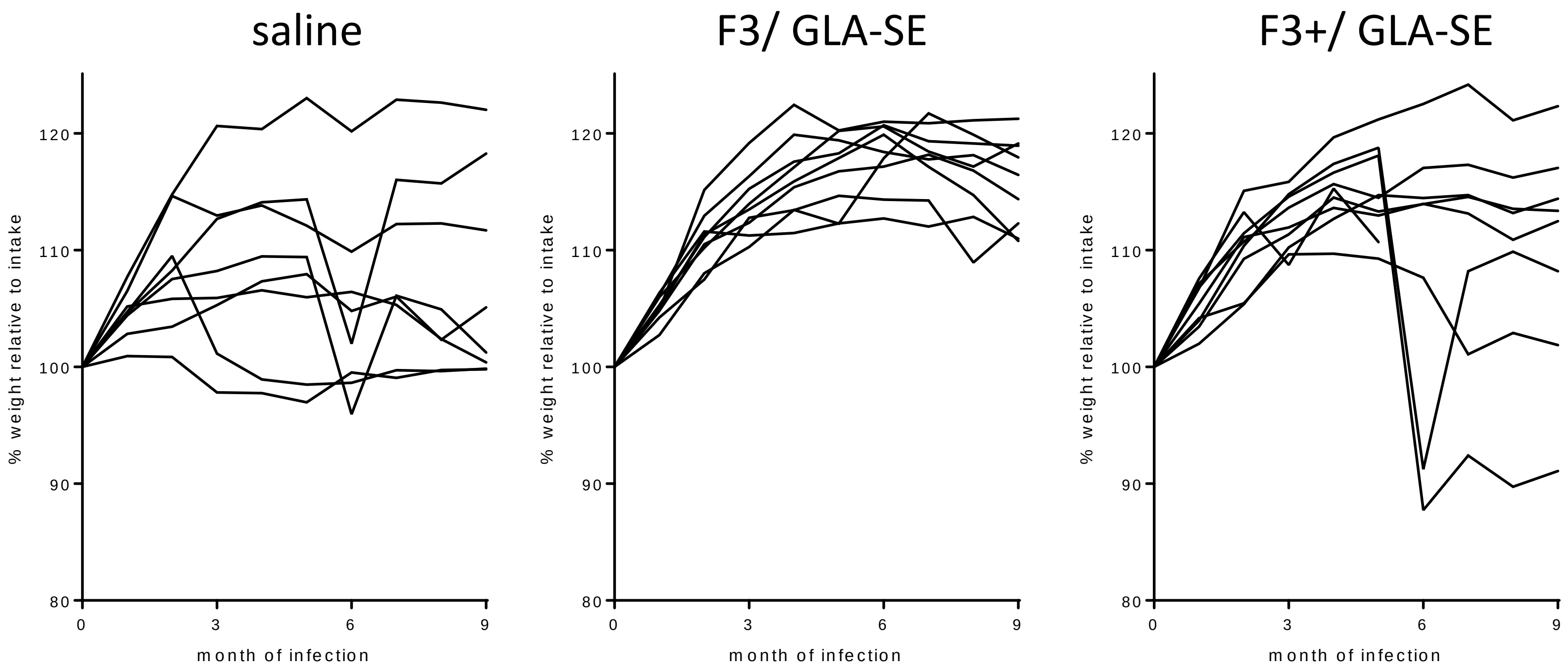

Supplementary Figure 3. Immunization with LEISH-F3/ GLA-SE or LEISH-F3+/GLA-SE reduces parasite burden in hamsters infected with *L. donovani* during sand fly blood meals. Hamsters were injected a total of 3 times with 5µg protein formulated with GLA-SE, then one month after the final immunization were infected by bites of *L. donovani*-infected sand flies, then monitored. Hamster weights were checked just prior to exposure to sand flies then monitored throughout the infection phase of the experiment. Each line depicts the relative weight of an individual hamster.
