# Supplementary material for: A defined subunit vaccine that protects against vector-borne visceral leishmaniasis
Source: NPJ Vaccines. 2017 Aug 21;2:23. doi: 10.1038/s41541-017-0025-5 (PMC5627294; doi:10.1038/s41541-017-0025-5)
Supplement: Supplementary file 4 — Supplementary Figure 4 [file 41541_2017_25_MOESM4_ESM.pdf]

Duthie et al., Suppl Figure 4

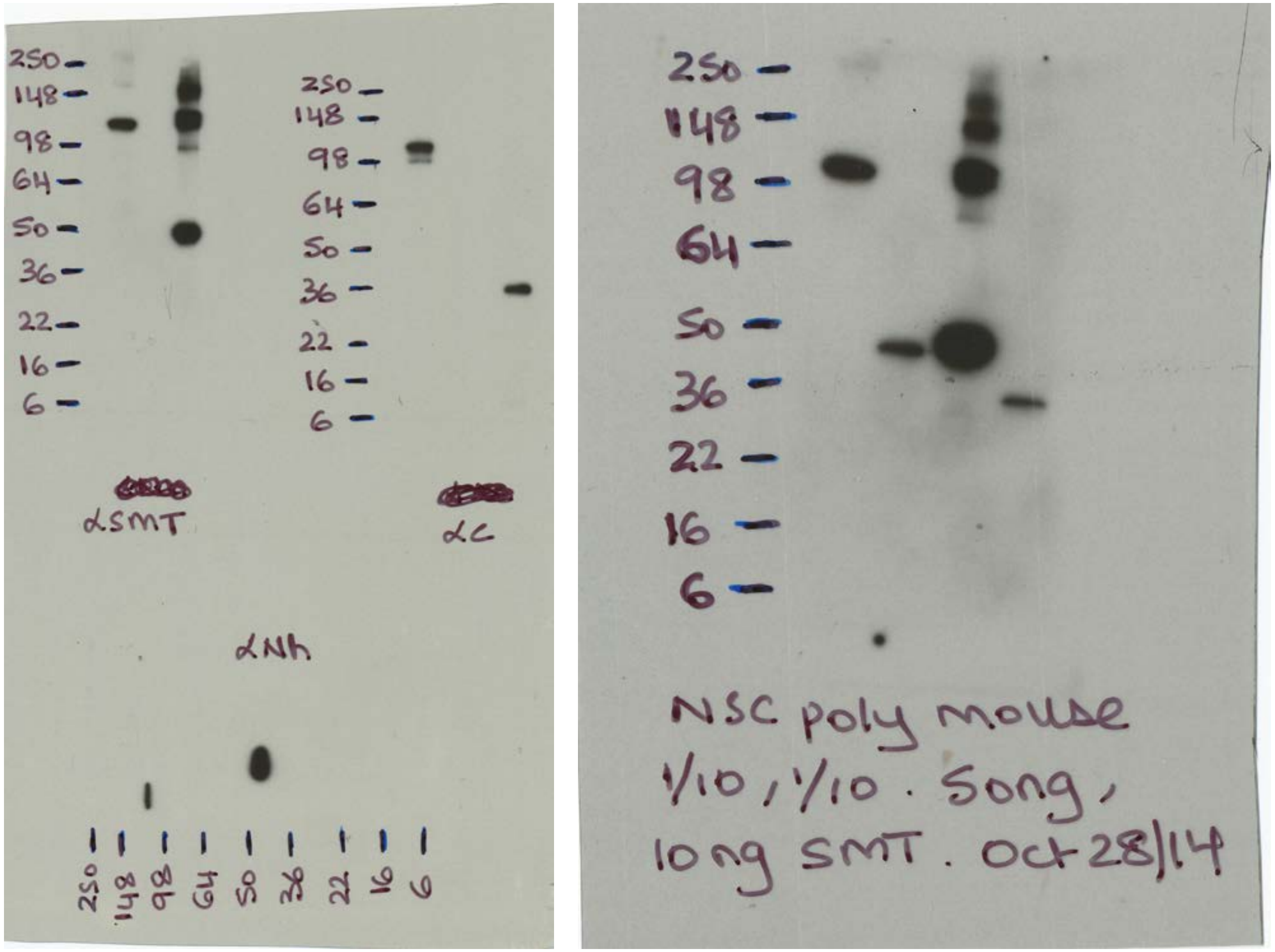

Supplementary Figure 4. Characterization of the LEISH-F3+ fusion protein by immunoblot. Recombinant LEISH-F3+ (lane 1), Nucleoside Hydrolase (NH; lane 2), Sterol-24-c-methyl-transferase (SMT; lane 3) or truncated Cysteine Protease B protein (ΔCPB; lane 4); were loaded at 100ng each lane into gels. Blots were developed with mouse polyclonal or monoclonal antibodies as indicated, derived from the same original gel.
